# Evaluation of the Effect of *Plectranthus amboinicus* L. Leaf Extracts on the Bacterial Antioxidant System and Cell Membrane Integrity of *Pseudomonas aeruginosa* PA01 and *Staphylococcus aureus* NCTC8325

**DOI:** 10.3390/pathogens12060853

**Published:** 2023-06-20

**Authors:** Sheeba Sawant, Timothy C. Baldwin, Oliwia Metryka, Ayesha Rahman

**Affiliations:** 1 Faculty of Science and Engineering, University of Wolverhampton, Wulfruna St., Wolverhampton WV1 1LY, UK; s.sawant2@wlv.ac.uk (S.S.); t.baldwin@wlv.ac.uk (T.C.B.); 2 Doctoral School, University of Silesia, Bankowa 14, 40-032 Katowice, Poland; oliwia.metryka@us.edu.pl; 3 School of Healthcare, College of Life Sciences, University of Leicester, Leicester LE1 7RH, UK

**Keywords:** catalase, lipid peroxidation, reactive oxygen species, cytoplasmic membrane, efflux pump inhibition, Indian borage, oxidative stress

## Abstract

*Plectranthus amboinicus* (Indian borage) has been extensively studied for its medicinal properties, which can be exploited to develop new antimicrobial therapeutics. The current study investigated the effect of *Plectranthus amboinicus* leaf extracts on the catalase activity, reactive oxygen species, lipid peroxidation, cytoplasmic membrane permeability, and efflux pump activity in *S. aureus* NCTC8325 and *P. aeruginosa* PA01. As the enzyme catalase protects bacteria against oxidative stress, disruption of its activity creates an imbalance in reactive oxygen species (ROS) levels, which subsequently oxidizes lipid chains, leading to lipid peroxidation. In addition, bacterial cell membranes are a potential target for new antibacterial agents, as efflux pump systems play a crucial role in antimicrobial resistance. Upon exposure of the microorganisms to Indian borage leaf extracts, the observed catalase activity decreased by 60% and 20% in *P. aeruginosa* and *S. aureus,* respectively. The generation of ROS can cause oxidation reactions to occur within the polyunsaturated fatty acids of the lipid membranes and induce lipid peroxidation. To investigate these phenomena, the increase in ROS activity in *P. aeruginosa* and *S. aureus* was studied using H_2_DCFDA, which is oxidized to 2′,7′-dichlorofluorescein (DCF) by ROS. Furthermore, the concentration of lipid peroxidation product (malondialdehyde) was assessed using the Thiobarbituric acid assay and was shown to increase by 42.4% and 42.5% in *P. aeruginosa* and *S. aureus,* respectively. The effect of the extracts on the cell membrane permeability was monitored using diSC3-5 dye and it was observed that the cell membrane permeability of *P. aeruginosa* increased by 58% and of *S. aureus* by 83%. The effect on efflux pump activity was investigated using Rhodamine-6-uptake assay, which displayed a decrease in efflux activity of 25.5% in *P. aeruginosa* and 24.2% in *S. aureus* after treatment with the extracts. This combination of different methods to study various bacterial virulence factors provides a more robust, mechanistic understanding of the effect of *P. amboinicus* extracts on *P. aeruginosa* and *S. aureus*. This study thus represents the first report of the assessment of the effect of Indian borage leaf extracts on bacterial antioxidant systems and bacterial cell membranes, and can facilitate the future development of bacterial resistance modifying agents derived from *P. amboinicus*.

## 1. Introduction

Plant-derived products have been used globally as medicines since ancient times. These natural medicines are known for their inhibitory properties against various pathogens and their role in boosting the host’s immunity is well established [1,2]. The modern era of medicine has increased the demand for the development of novel bioactive plant-derived compounds and the investigation of their inherent antimicrobial properties, as well as resistant modifying agents (RMA) that may also be present [3]. In recent years, the pharmaceutical industry has started to incorporate the use of phytochemicals in their research, as these substances may provide a major source of new drugs and slow down the emergence of antimicrobial resistance (AMR) [4,5].

AMR has become a major threat to public health and has been recently recognized by the World Health Organization as a potential problem in healthcare settings. Inappropriate prescribing, lack of proper drug regulatory systems, and limited diagnostic resources have worsened the situation in developing countries [6]. Bacteria manifest their resistance to antibiotics through different mechanisms. Some bacterial species are inherently resistant to certain classes of antibiotics. This causes all strains of a particular bacterial species to be resistant to all of the members of the same antibiotic class. Intrinsic resistance in Gram-negative bacteria is due to the presence of the outer lipopolysaccharide layer leading to reduced permeability and also due to an increase in efflux pump activity. For example, *Enterococci* are inherently resistant to aminoglycosides, lincosamides, and cephalosporins. Similarly, *Escherichia coli* is resistant to macrolides and *Klebsiella* spp. is resistant to ampicillin [1]. Another major concern is acquired resistance, where the primarily susceptible bacterial population becomes resistant to an antibiotic and proliferates [7,8]; for example, the acquired resistance exhibited by *Staphylococcus aureus* to methicillin [1].

*Pseudomonas aeruginosa* and *Staphylococcus aureus* are two of the major causes of acute nosocomial infections. These bacteria can survive treatment with antibacterial drugs because of their multi-drug resistant nature [2] and, thus, are included in the ESKAPE pathogens list (*Enterococcus faecium*, *S.* aureus, *Klebsiella pneumoniae*, *Acinetobacter baumannii*, *P.* aeruginosa, and *Enterobacter* species). These microbes have been placed on the priority list by WHO for the development of new antibiotics and other antimicrobial agents to combat AMR and, thus, were selected for use in the current study [9]. *P. aeruginosa* and *S. aureus* are known to exhibit resistance to antibiotics through limiting bacterial cell membrane permeability and efflux pump activity [10]. Furthermore, they use antioxidant enzymes such as catalases, which contribute to their survival under conditions of oxidative stress [11].

Under oxidative stress, O_2_ reduction processes generate reactive oxygen species such as hydroxyl radical (OH∙), superoxide anion (O_2_^−^), and hydrogen peroxide (H_2_O_2_). These ROS cause oxidative damage to the cellular macromolecules and may lead to cellular metabolic dysfunction. To overcome this threat, bacteria use defense and scavenging enzymes such as catalases, known as reactive oxygen species defense enzymes, that catalyze the dismutation of hydrogen peroxide [3]. As the key H_2_O_2_ scavenger enzyme, catalase plays a significant role in protecting bacteria against oxidative stress and retaining intracellular hydrogen peroxide concentrations [4]. Therefore, impairment in the catalase activity can create an imbalance in the intracellular concentration of H_2_O_2_. Another major effect of ROS activity is lipid peroxidation. Cell membranes consist of polyunsaturated fatty acids, which make them sensitive to radical damage. In addition, lipid peroxidation occurs when phospholipids interact with a ROS oxidizing agent. This phenomenon causes changes in the membrane structure by altering its fluidity and damaging the membrane integrity [5]. Furthermore, membrane integrity plays an important role in controlling cytoplasmic pH in bacteria. Bacterial cells maintain a pH gradient, which is higher inside than the outside of the cell, for cellular viability [6]. Efflux-pump-mediated resistance to multiple drugs has been a major concern due to limited treatment alternatives against bacterial infections. Efflux pumps decrease antibiotic accumulation inside the bacterial cells, which helps the bacterium to develop resistance against antibiotics [7]. Therefore, targeting these mechanisms by using *Plectranthus amboinicus* leaf extracts may help in controlling and treating infections caused by these microorganisms [3].

*Plectranthus amboinicus*, also known as Indian borage, belongs to the *Lamiaceae* family, and is a plant of medicinal interest due to its antimicrobial bioactivity. This plant grows well in tropical and sub-tropical areas and is indigenous to Asia, Africa, and Australia. The tissues of this species contain several volatile and non-volatile bioactive compounds that have been reported to exhibit a wide variety of therapeutic properties such as anti-inflammatory, antimicrobial, antitumor, anti-epileptic, antioxidant, larvicidal, and analgesic activities [8,9,10]. The leaves of Indian borage are traditionally used to treat colds, headache, skin diseases, constipation, and asthma. The leaves are aromatic and thus used as a flavoring agent while preparing traditional foods [1,2]. The phytochemical compositions and concentrations of the bioactive compounds may vary due to changes in geographical conditions, climate, soil, humidity, pH, and methods of extraction [11]. Some of the bioactive constituents found in Indian borage extracts are amino acids such as aspartic acid, serine, glutamic acid, and lysine. Flavonoids such as rutin, luteolin, and chrysoeriol and phenolic acids, for example gallic acids and rosmarinic acids, have also been isolated using Gas Chromatography−Mass Spectrometry analysis [12]. Essential oils extracted from the leaves of Indian borage have shown the presence of thymol, carvacrol, p-cymene, and eugenol, which contribute to the plant’s antimicrobial properties [2,13,14].

The aim of the current study was to investigate the effect of Indian borage leaf extracts upon bacterial catalase activity and the reactions leading to ROS generation and lipid peroxidation within the selected bacterial species. In addition, the bacterial membrane integrity was evaluated after exposure to the leaf extract. This research aimed to determine the suitability of Indian borage as a potential, commercially viable drug candidate, to meet the demand for cost-effective, safe, naturally derived, and antimicrobial agents. The global availability of this plant, ease of cultivation, and its unique antimicrobial properties, as demonstrated here and in previous studies, strongly indicate that this species represents a good candidate for commercial use as an antimicrobial agent and warrants further research and development.

## 2. Materials and Methods

### 2.1. Plant Material

The plant material used for the current study was *Plectranthus amboinicus (Lour.) Spreng*, also known as Indian borage (IB). For the study, botanically authenticated *Plectranthus amboinicus* plants were purchased from the National Herb Centre, Oxfordshire, United Kingdom, in September 2019, and were propagated from stem cuttings and grown in the glasshouse facility based at the University of Wolverhampton. Healthy leaves from the log stage of leaf development, measuring 20 mm (+/− 1 mm) in length, were subsequently harvested for the preparation of the leaf extract.

### 2.2. Extract Preparation

Here, 100 g of leaves harvested at the log phase of leaf development (20 mm +/− 1 mm in length) were macerated in 250 mL 70% acetone. Out of all of the solvent extracts used in our previous studies [15], the acetone extracts showed the highest antibacterial activity due to the high solubility of phenolic compounds. Therefore, 70% acetone was used as a solvent for extraction. These acetone leaf extracts were agitated using a magnetic stirrer, for 72 h, at 30 °C. The extracts were then filtered through Whatman filter paper No. 1 (Cytiva Whatman™, Thermo Fisher Scientific, Loughborough, UK) to obtain a particle-free filtrate. The solvent present in the extracts was subsequently evaporated in a rotary evaporator (Fisher Scientific, Loughborough, UK) under vacuum at 25 mmHg at 40 °C. Finally, the extracts were dispensed into 10 g aliquots and dissolved in 0.1% DMSO (Hybri-Max™, Sigma Aldrich, Gillingham, UK), to form stock solutions. According to the data obtained in our previous research, an MIC value of 25 mg/mL of leaf extract was used for both of the target bacterial species, as described below [15]. The MIC values used for ciprofloxacin were 0.5 µg/mL and 1 µg/mL against *S. aureus* and *P. aeruginosa,* respectively. All experiments were performed in triplicate, and the results shown represent the mean values of independent experiments.

### 2.3. Effect of IB Extracts on Catalase Activity in S. aureus NCTC8325 and P. aeruginosa PA01

Fresh colonies of *S. aureus* and *P. aeruginosa* were inoculated in 5 mL sterile tryptone soy broth (TSB) and followed by incubation at 37 °C in a shaker overnight to set up untreated culture controls. Similarly, fresh colonies of *S. aureus* and *P. aeruginosa* were grown in 1 mL of IB extracts (MIC) and 4 mL of tryptone soy broth overnight. Then, 100 μL of the respective test samples corresponding to O.D. of 0.1 was transferred into a test tube.

Subsequently, 100 μL of 1% Triton X-100 (Invitrogen^TM^, Thermo Fisher Scientific, Loughborough, UK) and 100 μL of 30% (*v*/*v*) H_2_0_2_ were added to the tube. After mixing thoroughly, the tubes were incubated at room temperature for 5 min. After the reaction stopped and the height of the foam remained constant for 15 min, it was measured in cm using a ruler [16].

#### Calculations

For each sample, the height of the foam was expressed as percentage change using the following formula.

$$\%\;change\;=\;\left[\begin{array}{c} \frac{\left\{height\;of\;foam\;\left(exposed\;cells\right)\;-\;height\;of\;foam\;\left(unexposed\;control\right)\right\}}{}\\ height\;of\;foam\;\left(unexposed\;control\right) \end{array}\right]\;*\;100$$

### 2.4. Lipid Peroxidation Assay

The level of lipid peroxidation was assessed by the concentration of malondialdehyde using thiobarbituric acid (TBA) [17]. In this method, there is the formation of a colored adduct due to the reaction between TBA and malondialdehyde (lipid peroxidation products) in an acidic environment, at an elevated temperature. The resulting adduct has a red coloration and its concentration is determined by the amount of absorbed light at 535 nm. In this experiment, we compared the untreated culture sample and 1% DMSO, ciprofloxacin, and Indian borage-treated culture samples to examine the level of lipid peroxidation upon exposure to these agents. Fresh colonies of *S. aureus* and *P. aeruginosa* were grown in 1 mL of IB extracts and 4 mL of tryptone soy broth (Millipore Merck, Watford, UK) overnight. After 24 h of incubation, 1 mL of the respective test samples, 1 mL of thiobarbituric acid (Sigma-Aldrich, Gillingham, UK), and 1 mL of trichloroacetic acid (Sigma-Aldrich, Gillingham, UK) were added into the screw cap test tube. Two controls were prepared, one with 1 mL of water instead of the test sample, and another with 1 mL water instead of TBA. After mixing the contents well, the tubes were placed in a water bath at 100 °C for 10 min. After cooling, the tubes were centrifuged at 5000 rpm for 20 min. After centrifugation, the supernatant was measured at 535 nm against the first control as it was devoid of a test sample and only consisted of TBA. Furthermore, the values obtained were subtracted from the second control. The final value measures the amount of malondialdehyde. The concentration of malondialdehyde was calculated based on the millimolar absorbance coefficient equal (E_0_) to 156 L/cm^−1^ × mmol^−1^ using the equation shown below and as described in Supplementary Tables S1.1 and S1.3.

(1) $$Malondialdehyde\;concentration\;\left(mM\right)\;=\;\frac{\left\{Sample\;absorbance\;\left(exposed\;cells\right)\;-\;Blank\right\}}{E_{0}\;(1561)}$$

### 2.5. Measurement of Reactive Oxygen Species Concentration with 2′,7′-Dichlorodihydrofluorescein Diacetate (H_2_DCFDA)

First, 1 mL of overnight cultures of *S. aureus* and *P. aeruginosa* were suspended in 4 mL of PBS (pH 7.2), respectively, with 1 ml of IB extracts and incubated at 37 °C for 1 h on a shaker. Similarly, the following blank controls were prepared; one with IB extracts and without the bacterial cells and another one with bacterial cells without IB extracts. After 1 h, 4 mM H_2_DCFDA (Thermofisher scientific, Swindon, UK) at 200 µL was added to the test samples and incubated for 30 min. The fluorescence was measured in an Clariostar Plus multi-mode plate reader (BMG Labtech, Offenburg, Germany) at an excitation wavelength of 485 nm and an emission wavelength of 530 nm. The intracellular ROS concentration was estimated according to the Alpaslan method [11] and expressed as AU·CFU mL^−1^, where AU means the absorbance unit.

### 2.6. Evaluation of Cell Membrane Integrity as a Potential Antimicrobial Target

#### 2.6.1. Cytoplasmic Membrane Permeability Assay

A cytoplasmic membrane permeability assay was carried out using the method of Nusslein et al. [18]. The membrane potential sensitive cyanine dye, 3,3′-Dipropylthiadicarbocyanine iodide (diSC3-5) was used to determine the bacterial cytoplasmic membrane permeability. DiSC3-5 dye is distributed between the surrounding medium and the bacterial cells, depending on the membrane potential gradient. Once the dye is inside the membrane, it is known to aggregate and self-quench. Due to the addition of membrane-permeabilizing agents, the dye was released, and the fluorescence overtime was monitored. *S. aureus* and *P. aeruginosa* cells were grown overnight in 20 mL TSB at 37 °C in a shaker. The cells were collected after 24 h by centrifugation at 3000 rpm for 20 min. The cells were washed with 5 mL of 5 mM glucose buffer (pH 7.2), 5 mM HEPES, and resuspended in PBS (pH 7.2). The cells were incubated in 1 μM diSC3-5 for 1 h, for maximal uptake of dye. Then, 100 mM KCl (100 μL) was added to the cells to equilibrize the cytoplasmic and external K+ ion concentrations. Then, 100 μL of IB extract was added and incubated for 30 min. The fluorescence was observed at an excitation wavelength (622 nm) and an emission wavelength (670 nm). Then, 1% DMSO was used as a positive control and monitored for dye release and acetone was used as a negative control as the extracts were dissolved in it. The monitoring period was for 1 h at 10 min interval. Then, 750 μL sample aliquots were taken at 10 min intervals and placed in Eppendorf tubes with 750 μL of HEPES buffer. The suspensions were then centrifuged, and the supernatants were collected for measuring fluorescence using the spectroflourophotometer (BMG Labtech, Offenburg, Germany).

#### 2.6.2. Rhodamine 6-G Uptake Assay

Rhodamine 6-G uptake assay was done using the method of Maesaki et al. [19]. *S. aureus* and *P. aeruginosa* were cultured overnight in St. TSB at 37 °C in a shaker. After 24 h of incubation, the cells were centrifuged using 4000 rpm for 5 min and washed twice with PBS (pH 7.2). The cells were centrifuged again and re-suspended in 15 mL PBS containing 10 mM NaN_3_. Then, 10 µm Rhodamine-6-G (Thermo fischer scientific, Swindon, UK) at 300 µL was added and cells were placed in an incubator for 1 h. The cell suspension was centrifuged at 4000 rpm for 10 min and resuspended in 30 mL PBS containing 1 M glucose. The cells were divided into 1 mL aliquots in eppendorfs and IB extracts (500 µL) were added to the cells. The samples were placed in shaking incubator for 30 min at 37 °C. The cell suspension was centrifuged at 4000 rpm for 10 min, and the supernatant was discarded. The remaining pellet was suspended in 1 mL 0.1 M glycine HCl (pH 3) and placed in the shaker overnight. After 24 h, the cells were centrifuged for 10 min at 4000 rpm, and the supernatant was collected for measuring absorbance at 527 nm using an ELISA plate reader (Spectrostar^nano^, BMG Labtech, Offenburg, Germany). A standard plant-based efflux pump inhibitor, reserpine (Sigma-Aldrich, Gillingham, UK), was used as a positive control.

### 2.7. Statistical Analysis

Analysis of variance (ANOVA) was used to determine the significance (*p* Value) of the data obtained from the lipid peroxidation assay, ROS activity, and evaluation of the cell membrane integrity using Prism-GraphPad Version 9.5.1 by Dotmatics, Boston, USA.

## 3. Results

### 3.1. Catalase Activity

**Figure 1:**
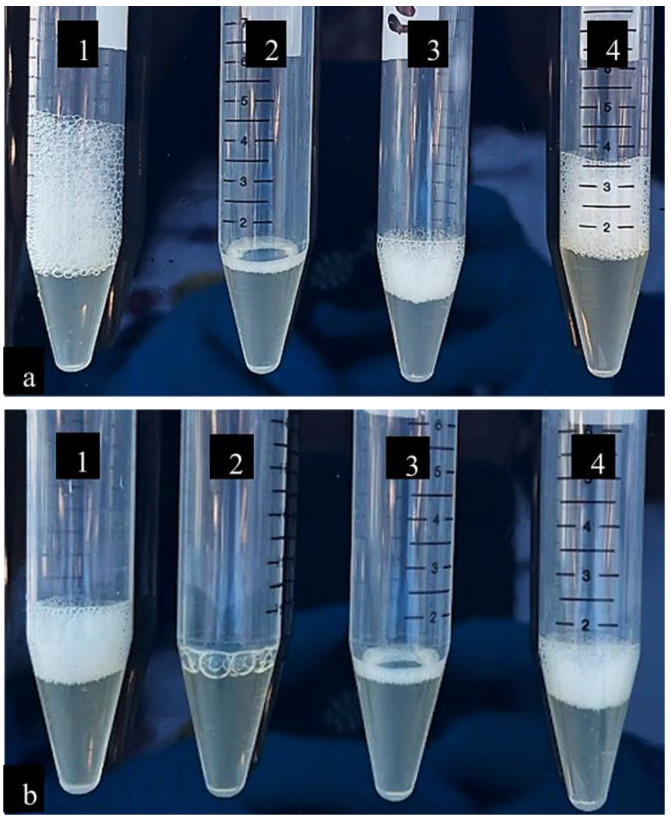
(**a**) Effect of Indian leaf borage extracts on catalase activity in *P. aeruginosa*. (1) Untreated cells. (2) Ciprofloxacin-treated cells, drug control. (3) IB-extract-treated cells. (4) 1% DMSO-treated cells, solvent control. (**b**) Effect of Indian borage leaf extracts on catalase activity in *S. aureus*. (1) Untreated cells. (2) Ciprofloxacin-treated cells, drug control. (3) IB-extract-treated cells. (4) 1% DMSO-treated cells, solvent control.

The catalase activity of *P. aeruginosa* and *S. aureus* was visually determined using the hydrogen peroxide method. The height of foam (cm) was measured to calculate the percentage change in catalase activity. Table 1 displays the effect of IB extracts on the catalase activity in *P. aeruginosa*. IB-treated cells revealed a 60% decrease in catalase activity as the height of foam reduced considerably, from 2.5 cm in the control (untreated cells) to 1 cm in the IB-treated cells (Figure 1a: 3). Ciprofloxacin was used as a drug control and showed an 88% reduction in catalase activity with 0.3 cm of foam height (Figure 1a: 2). Cells were treated with 1% DMSO (Figure 1a: 4) as a solvent control, and displayed 2 cm foam height and a 20% decrease in activity.

The effect of IB extracts on catalase activity in *S. aureus* is presented in Table 2 IB-treated cells displayed a 50% decrease in catalase activity as the height of foam reduced from 1 cm (untreated cells) to 0.5 cm (Figure 1b: 3). Cells treated with ciprofloxacin (Figure 1b: 2) displayed no foam. No change in foam height was observed in cells treated with 1% DMSO (Figure 1b: 4). The results revealed the range of catalase activity was affected in *P. aeruginosa* and *S. aureus* grown in IB extracts, in comparison with its effect on the ciprofloxacin and 1% DMSO-treated samples.

The images above display the height of foam formed during the hydrogen peroxide-catalase reaction, where hydrogen peroxide was reduced to water and oxygen. The height of the foam was measured to quantify the effect of the leaf extracts on the catalase activity. *P. aeruginosa* and *S. aureus* untreated samples displayed 2.5 cm and 1 cm of foam, respectively. In *P. aeruginosa*, 1% DMSO-treated samples showed 2 cm of foam, followed by IB-treated samples with 1 cm and the lowest height of foam was seen in the ciprofloxacin-treated sample at 0.3 cm. In *S. aureus*, 1% DMSO showed 1 cm foam, followed by the IB-treated samples with 0.8 cm and ciprofloxacin-treated samples with 0.5 cm of foam height.

### 3.2. Lipid Peroxidation

In *P. aeruginosa*, upon exposure to Indian borage leaf extracts, the resulting malondialdehyde concentration was 42.38% (813 nm) more than the culture control (571 nm). 1% DMSO was used as a solvent control and resulted in a 2% (560 nm) decrease in malondialdehyde concentration. However, ciprofloxacin, the antibiotic control, was able to increase lipid peroxidation products by 51.31% (864 nm).

Similarly, in *S. aureus*, samples exposed to Indian borage leaf extracts induced a 42.5% (365.1 nm) increase in lipid peroxidation products, in comparison with the control sample (256 nm). For antibiotic control, ciprofloxacin displayed a 57% (403.5 nm) rise in malondialdehyde concentration. In contrast, 1% DMSO-treated samples showed a 10% (230.6 nm) drop in malondialdehyde concentration.

From theresults shown in Table 3 and the ANOVA analysis, it was established that Indian borage leaf extracts were able to induce a significant (*p* < 0.001) increase in the levels of lipid peroxidation products in both bacterial species.

### 3.3. Reactive Oxygen Species

**Figure 2:**
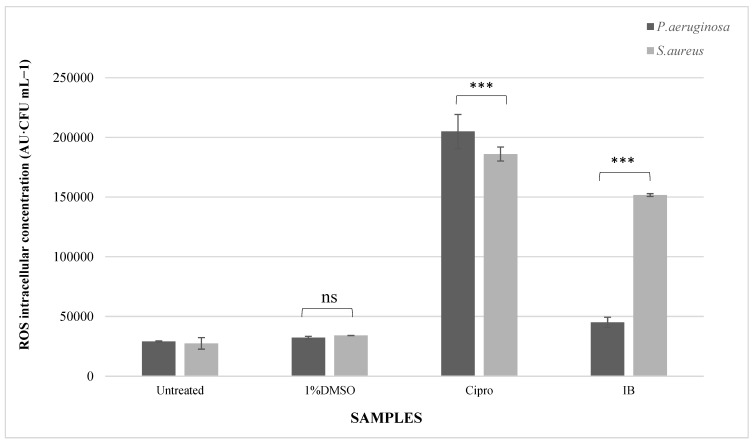
Effect of IB leaf extracts on ROS concentration in *P. aeruginosa* and *S. aureus*. Untreated: untreated culture control; 1% DMSO: solvent control; Cipro: ciprofloxacin, drug control; IB: Indian borage leaf extract. The bar graph indicates the reactive oxygen species (ROS) intracellular concentrations in AU·CFU mL^−1^ in bacterial cells after exposure to different compounds. The vertical bars denote the mean values based on three replicates with error bars representing the standard deviation. The data labels above the bar graphs denote the ROS intracellular concentration in AU·CFU mL^−1^. The untreated *P. aeruginosa* samples displayed 28,975 AU·CFU mL^−1^ of ROS concentration, the concentration changed to 32,225 AU·CFU mL^−1^, 204,943.6 AU·CFU mL^−1^, and 45,060.6 AU·CFU mL^−1^ after exposure to 1% DMSO, cipro, and IB extracts, respectively. Similarly, the untreated *S. aureus* samples showed 27,375.33 AU·CFU mL^−1^. However, after 1% DMSO, Cipro and IB treatment, the ROS concentration changed to 34,006.3 AU·CFU mL^−1^, 186,044.3 AU·CFU mL^−1^, and 151,678.6 AU·CFU mL^−1^, respectively. The *p* value denoted by asterisks (***) reveals that ciprofloxacin and IB extracts had a significant (*p* < 0.001) effect on the ROS intracellular concentration levels in comparison with the untreated control. The non-significant effect of 1% DMSO on ROS activity is indicated by ns.

To determine the effect of the IB leaf extracts on ROS induction in *P. aeruginosa* and *S. aureus*, H_2_DCFDA dye was used to measure the ROS intracellular concentration. In *P. aeruginosa*, ciprofloxacin was shown to significantly increase (*p* < 0.001) ROS activity (204,943.6 AU·CFU mL^−1^), parallel to IB leaf extracts, which induced ROS generation (45,060.6 AU·CFU mL^−1^) by 55.51%, as shown in Figure 2. Similarly, 1% DMSO, the solvent control, showed an 11.21% increase in ROS activity (32,225 AU·CFU mL^−1^). All of the treated samples displayed a significant increase (*p* < 0.001) in comparison with the control.

In *P. aeruginosa*, the ciprofloxacin-treated samples displayed a marked increase in ROS activity (204,943.6 AU·CFU mL^−1^). Similarly, IB leaf extracts induced a significant (*p* < 0.001) rise in ROS generation, of 45,060.6 AU·CFU mL^−1^. Furthermore, 1% DMSO showed a 36% increase in ROS generation, which was not statistically significant in comparison with the control and IB leaf extracts. The ROS induction by IB leaf extracts was much higher in *S. aureus* in comparison with *P. aeruginosa.*

From these results and the ANOVA analysis, it was evident that the Indian borage leaf extracts were able to induce ROS generation in *P. aeruginosa,* as well as *S. aureus* samples at statistically significant (*p* < 0.001) levels.

### 3.4. Evaluation of Cell Membrane Integrity as a Potential Antimicrobial Target

#### 3.4.1. Cytoplasmic Membrane Permeability Assay

**Figure 3:**
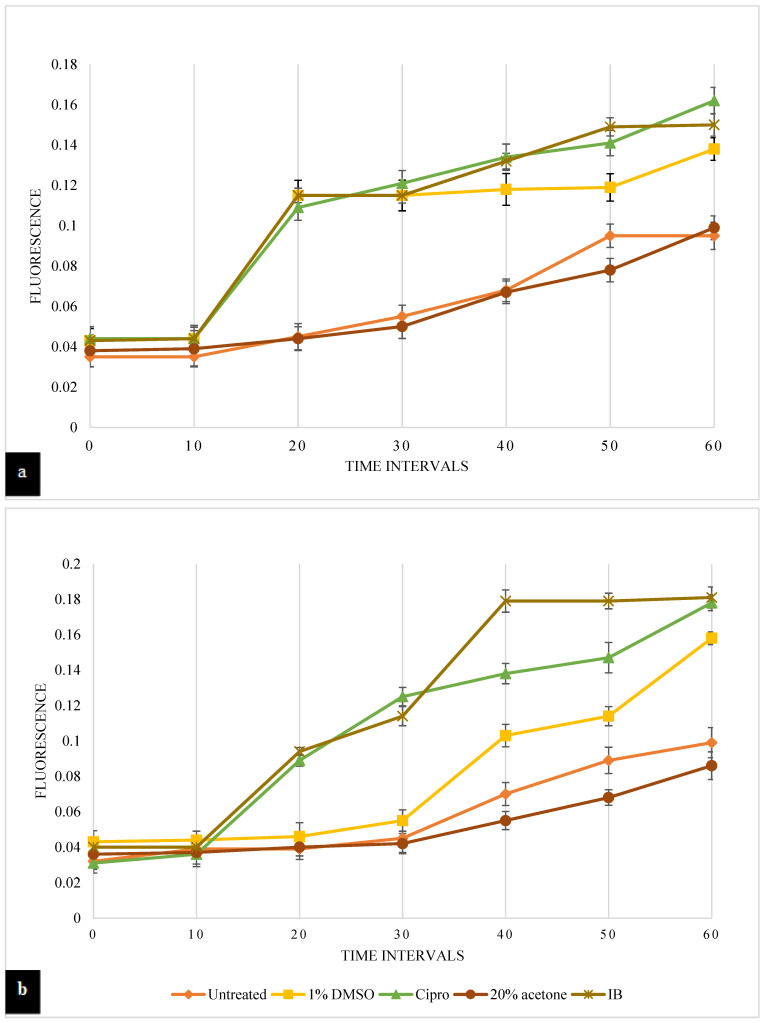
(**a**) Effect of IB extracts on cytoplasmic membrane integrity in *P. aeruginosa*. (**b**) Effect of IB leaf extract on cytoplasmic membrane integrity in *S. aureus*. Fluorescence: The fluorescence is observed at an excitation wavelength (622 nm) and an emission wavelength (670 nm). Untreated: untreated culture control; 1% DMSO: positive control; Cipro: ciprofloxacin, drug control; 20% acetone: solvent control; IB: Indian borage leaf extract. The graph indicates the fluorescence of diSC3-5 dye released at different time intervals after exposure to different treatments in *P. aeruginosa* (**a**) and *S. aureus* (**b**). In *P. aeruginosa*, the cipro and IB-treated samples show the highest dye release of 71% with 0.162 O. D and 58% with 0.15 O. D at 60 min, respectively, followed by 1% DMSO with 45% at 0.138 O. D and 20% acetone with 4.2% at 0.099 O.D. In *S. aureus*, the IB- and cipro-treated samples displayed the highest dye release of 83% with 0.181 O. D and 78% with 0.178 O. D at 60 mins, respectively, followed by dye release of 59% with 0.158 O. D of 1% DMSO-treated samples at 60 min.

As seen in Figure 3a, Gram-negative *P. aeruginosa* was treated with IB leaf extract, and it was observed that the addition of IB leaf extracts led to an increase in fluorescence from 0.043 O. D at 0 min to 0.15 O. D at 60 min, thus presenting 58% of the dye release due to the dissipating membrane potential caused by exposure to the leaf extract. Dye release of 45% was seen over time in the presence of 1% DMSO, which was used as a positive control. The drug control (ciprofloxacin) displayed a maximum dye release of 71% at 60 min. Contrary to which, a 4.2% dye release was observed in the presence of 20% acetone, which was used as a solvent control.

In *S. aureus*, 83% of dye release was observed at 60 min in the presence of IB leaf extracts, as the fluorescence increased from 0.040 at 0 min to 0.181 at 60 min (Figure 3b). Furthermore, 1% DMSO showed a surge in release of diSC3-5 dye at 40 min (0.103 O.D.) and 59% dye release at 60 min. Ciprofloxacin led to a 78% release of dye at the end of the 60 min monitoring period. However, there was no evident increase in dye release seen in the presence of 20% acetone.

However, IB leaf extracts were observed to be more potent as cytoplasmic membrane permeability enhancers against *S. aureus* (83%) compared with *P. aeruginosa* (58%). Similarly, *S. aureus* membranes were shown to be more sensitive to 1% DMSO compared with those of *P. aeruginosa*. However, the presence of ciprofloxacin displayed a permeability enhancing effect on both of the bacterial membranes.

#### 3.4.2. Rhodamine-6-G Uptake Assay

**Figure 4:**
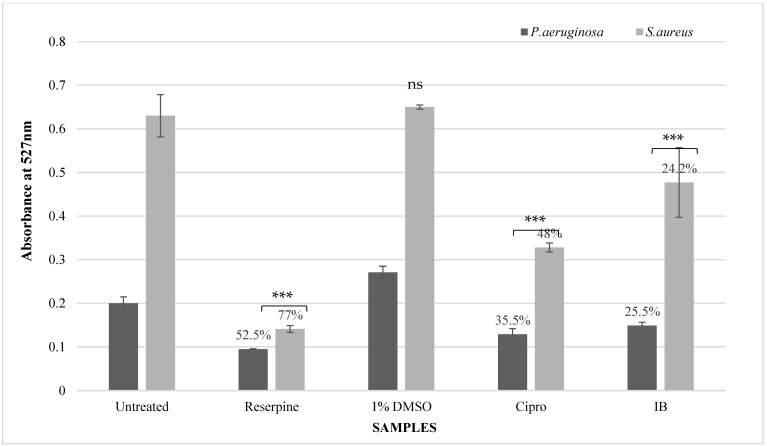
Effect of IB leaf extracts on efflux pumps in *P. aeruginosa* and *S. aureus* evaluated using the R-6-G uptake assay. Untreated: untreated culture control; Reserpine: standard efflux pump inhibitor control; 1% DMSO: solvent control; Cipro: ciprofloxacin, drug control; IB: Indian borage leaf extract. The bar graph indicates the efflux pump inhibition (EPI) activity by comparing the absorbance of Rhodamine-6-G dye after exposure to different compounds. Data labels above the bar graphs denote % efflux pump inhibition. In *P. aeruginosa*, reserpine-treated samples showed a maximum efflux pump inhibition of 52.5% followed by ciprofloxacin at 35.5% and IB leaf extracts at 25.5%. In *S. aureus*, reserpine-treated samples showed 77% of EPI activity, followed by 48% and 24.2% of EPI activity by the ciprofloxacin and IB-treated samples. The *p* value is denoted by asterisks in the figure. The effect of IB leaf extracts on efflux pumps in *P. aeruginosa* showed a significant (*p <* 0.001) impact on the EPI activity. Similarly, in *S. aureus*, a significant (*p <* 0.05) effect, was recorded on the EPI activity after exposure to the IB leaf extracts. The *p* value denoted by asterisks (***) reveals that the ciprofloxacin and IB extracts had a significant (*p* < 0.001) effect on the EPI activity in comparison with untreated control. The non-significant effect of 1% DMSO on the EPI activity is indicated by ns.

As shown in Figure 4, reserpine was shown to exhibit the highest efflux pump inhibition (EPI) of 52.5% and 77% in *P. aeruginosa* and *S. aureus*, respectively. In *P. aeruginosa*, the absorbance of rhodamine-6-G dye in untreated samples was 0.2 O. D, which was observed to decrease to 0.149 O. D after treatment with IB leaf extract. Similarly, in *S. aureus*, the absorbance dropped from a 0.63 O. D in the untreated samples to 0.477 O. D in the samples treated with the IB extract. Treatment with the IB extract produced a similar effect on the bacterial EPI activity, in both *P. aeruginosa* and *S. aureus*, with a 25.5% and 24.2% inhibition observed in each species, respectively. Furthermore, the drug control, ciprofloxacin, produced a 35.5% and 48% reduction in EPI activity in *P. aeruginosa* and *S. aureus*, respectively. Although the IB leaf extract was shown to inhibit efflux pump activity, the reduction in activity was lower than the standard plant-based EPI, reserpine. These observations revealed that the IB leaf extracts were equally potent against *P. aeruginosa* and *S. aureus*. However, according to ANOVA analyses, the effect on the EPI in *P. aeruginosa* was more statistically significant (*p <* 0.001) than that recorded for *S. aureus* (*p* < 0.05).

## 4. Discussion

*Plectranthus amboinicus* is known to contain a variety of phytochemicals that possess medicinal properties [13,14,15]. In the present study, we examined the effect of Indian borage leaf extracts on a range of bacterial virulence factors in order to better understand the target mechanism of the extracts.

Many pathogens are known to produce catalase enzymes to protect themselves from hydrogen peroxide, a defense mechanism commonly used by the host’s immunity. Previous studies have reported that catalase-deficient mutant pathogens are more sensitive to oxidative stress and attack by the host immune system [20,21]. Our investigation on the effect of IB leaf extracts on catalase activity in *P. aeruginosa* and *S. aureus* has provided a deeper insight into the inherent mechanisms of bacterial pathogenicity and resistance to oxidative stress. In the current study, catalase activity was measured by the height of the trapped oxygen gas (foam) as seen in Figure 1. No reagents were used to end the reaction, and the generation of oxygen bubbles naturally ceased within 5 min. It was observed that in *P. aeruginosa*, the IB-treated samples displayed a 60% decrease, whereas in *S. aureus*, a 50% decrease in catalase activity was observed. As there is currently no literature available describing the mechanistic action of plant extracts upon bacterial catalase activity, it could be anticipated that the decrease in enzymatic activity may be due to the presence of phytochemicals. Phytochemicals such as thymol, carvacrol, p-cymene, and other phenolic acids are known to possess antibacterial properties and to inactivate bacterial enzyme systems as one of their potential modes of action [15,22].

**Figure 5:**
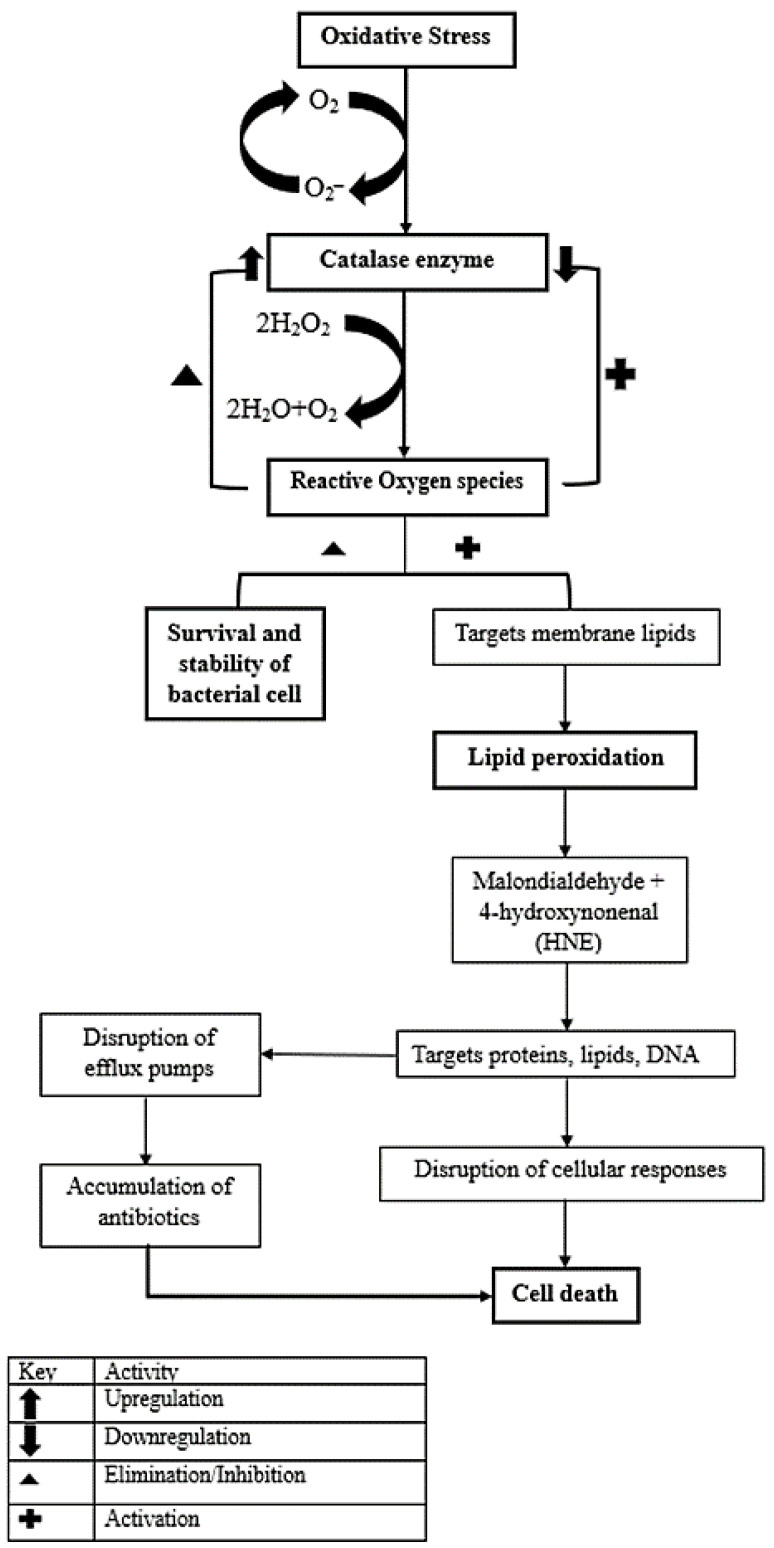
Schematic presentation of the effect of *P. ambioincus* extracts on *P. aeruginosa* and *S. aureus* virulence factors. Oxidative stress occurs due to an imbalance in redox status. Reactive oxygen species are continuously generated in aerobic organisms. Catalases are involved in ROS elimination by catalyzing the conversion of hydrogen peroxide to water and oxygen. Improved enzymatic activity of catalases, decreases the accumulation of ROS, and helps in the survival and stability of the bacterial cells. However, a decline or disruption in the catalase activity increases ROS concentration, which attacks membrane lipids causing lipid peroxidation. The accumulation of lipid peroxidation by products such as malondialdehyde and HNE target lipids, DNA, and proteins such as bacterial transport protein, which causes a disruption or reduction in efflux pumps activity and helps in accumulating antibiotics within the cells. Ultimately, this leads to a disruption in cellular responses and causes cell death.

Furthermore, catalase produced under conditions of oxidative stress can reduce lipid peroxidation [23,24]. However, in this study, the inhibition or disruption of the catalase enzyme activity may have caused a surge in reactive oxygen species and resulted in an increase in lipid peroxidation. In our study, lipid peroxidation was assayed by measuring the concentration of malondialdehyde using thiobarbituric acid, as shown for *P. aeruginosa* in Table S1.1 and for *S. aureus* in Table S1.2. IB-treated samples indicated an increase in lipid peroxidation of 42.38% and 42.5% in *P. aeruginosa* and *S. aureus*, respectively. Similarly, the reactive oxygen species intracellular concentration was also shown to increase in the presence of the IB extract. Figure 2 displays the observed increase in ROS activity in *P. aeruginosa* (45,060.6 AU·CFU mL^−1^) and *S. aureus* (151,618.6 AU·CFU mL^−1^) after treatment with the IB leaf extract. A possible explanation for these data may be that catalase activity is critical for oxidative stress resistance and reducing the level of lipid peroxidation. However, in this case, the inactivation of the catalase enzyme may cause an upregulation of ROS generation and thus lead to an increase in lipid peroxidation (Figure 5). These reactive species are known to chemically react with proteins, nucleic acids, lipids, and other cellular biomolecules, thus causing alterations and oxidative modifications that damage bacterial cellular activities [25]. As seen in Figure 2, Gram-positive bacteria produce more ROS than the Gram-negative bacteria compared with their controls; the specific factors contributing to this trend are yet to be determined.

Previous reports have investigated the metabolic importance of alkyl hydroperoxide reductase subunit C (AhpC), which is crucial for the detoxification of ROS in bacterial cells, and thus enables bacteria to survive under conditions of environmental stress [26]. Inactivation/inhibition of this enzyme results in an increase in lipid hydroperoxides, thus leading to the production of ROS and the induction of lipid peroxidation [23]. These lipid hydroperoxides can also inactivate catalase by the generation of catalytically incompetent compound II species and thereby result in lipid peroxidation [23]. These possibilities may explain the observed results, as well as the effects of Indian borage leaf extracts on the bacterial antioxidant systems.

In the study presented, the effect of the Indian borage leaf extracts on bacterial cell membrane integrity was investigated using two methods. The first method consists of using diSC3-5 dye to study the membrane permeabilizing properties of IB leaf extracts. The cytoplasmic membrane permeability of *P. aeruginosa* and *S. aureus* was affected after IB leaf extract treatment. *S. aureus* was seen to be more susceptible to the IB leaf extract with 83% dye release in comparison with *P. aeruginosa* with 58% dye release at 60 min (Figure 3). In *S. aureus,* the membrane permeabilizing activity of the IB leaf extract (83%) was higher than that recorded when using ciprofloxacin (78%). The contrast in susceptibility pattern between *P. aeruginosa* and *S. aureus* could be due to the differences in cell wall molecular architecture observed in the Gram-negative and Gram-positive bacteria. Gram-positive bacteria such *S. aureus* are easier to penetrate due to a single layer membrane and, thus, are more sensitive to antimicrobial agents; however, Gram-negative bacterial species have, in addition to a cell wall, an outer lipopolysaccharide layer, which makes them more resistant to antibiotics [27,28,29]. Previous studies have reported that upon treatment with Indian borage extracts, *B. cereus* and *Y. enterocolitica* exhibited degradation and rupture of their cell walls. Additionally, the extracts caused a loss of uniformity and smoothness of the treated cell walls, which was observed using scanning electron micrographs. The antibacterial activity of Indian borage extracts was attributed to the presence of flavonoids, terpenoids, alkaloids, and phenols, which target bacteria by causing cell wall degeneration and genetic material degradation [30].

The IB leaf extract contains essential oils that are known to affect the bacterial plasma membrane and inhibit the respiratory chain and energy production, thus leading to a weakening of the bacterial cell membrane [15]. Phytochemicals such as thymol and carvacrol present in the IB leaf extract may act as membrane permeabilizers and ally with fatty acids to enhance the cytoplasmic membrane permeability [22,31,32,33]. These bioactive components may also trigger lipid perturbation in the bacterial plasma membrane [34]. Saponins and glycosides present in Indian borage have high membrane penetrating abilities and are currently being studied as an antimicrobial alternative to antibiotics [35]. These membranotropic phytochemicals found in IB leaf extracts may exhibit their membrane permeabilizing properties through (i) binding to lipid components, (ii) disrupting the membrane protein activity, (iii) the formation of membrane microdomains, or (iv) altering the membrane fluidity [36,37]. Another possibility that may result in the distortion of the bacterial membrane could be the production of ROS, as shown in Figure 2. As shown in this figure, ROS generation was higher in *S. aureus,* which could lead to bacterial cell lysis [38], which would explain the high cytoplasmic membrane permeability observed in *S. aureus*.

The second method used to evaluate membrane integrity was the rhodamine-6-G (R-6-G) uptake assay (Figure 4). R-6-G, a fluorescent dye, acts as a substrate for bacterial efflux pumps and thus accumulates within these cells. Efflux pump inhibition prevents the release/egress of the dye. Hence, this phenomenon was used to study the EPI activity of the IB extract against *P. aeruginosa* and *S. aureus*. The plant-based EPI, reserpine, was used as the positive control in these experiments and displayed the highest EPI activity of 52% and 77% against *P. aeruginosa* and *S. aureus*, respectively. The IB extract showed a 25.5% and 24.2% EPI activity against *P. aeruginosa* and *S. aureus*, respectively. The EPI activity of the IB leaf extract could be further enhanced by isolating and identifying the EPI compounds present in the extract.

Many phytochemicals such as reserpine, act as a resistance modifying agents (RMA) [39,40]. This compound is a plant-derived alkaloid isolated from *Rauvolfia vomitoria* [41]. The flavonolignan 5-methoxyhydnocarpin and the diterpene totarol isolated from *Chamaecyparis nootkatensis* are reported to inhibit NorA efflux pump activity in *S. aureus* [40]. The exact mechanism for efflux pumps inhibition is still unknown; however, it is proposed that phytochemicals interact with these pumps and inactive them. Another possibility is the synergistic interactions of phytochemicals and the antibiotics, which lead to energy depletion within the cell and to the formation of a complex to facilitate its entry into the cells. The large size of the complex or the phytochemical molecules can block the efflux pumps and thus restore the intracellular concentration of the antibiotics within the cells. In a previous study to improve membrane permeability, caffeic acid and epicatechin 3-gallate were used to break down the oily outer membrane of Gram-negative bacteria such as *Salmonella enterica sv. typhimurium* and *E. coli*. Consequently, the combination of these chemical compounds with hydrophobic antibiotics such as rifampicin and erythromycin increase the penetration rate within the cells, which otherwise fail to reach the inside of bacteria, thus enhancing its antimicrobial action against bacteria [42,43]. Hence, the isolation of the phytochemicals present in the IB leaf extract, which are responsible for its EPI activity, could lead to the development of additional plant-based efflux pump inhibitors. Such inhibitors could potentially reduce the bacterial intrinsic and acquired resistance to antibiotics, and thereby contribute to the control of current and emerging antibiotic resistant bacterial strains [44].

This study represents the first report on the effect of Indian borage leaf extracts on the bacterial antioxidant system. The data presented have demonstrated that leaf extracts of *Plectranthus amboinicus* can inhibit a number of major bacterial virulence/resistance factors, including cell permeability, and efflux pumps in the selected target species. As such, this extract represents a useful antimicrobial agent worthy of further research and development.

## 5. Conclusions

The diversity of bioactive components found in plants is a sustainable and potential source of antimicrobials and resistance modifying agents. The development of antibiotics usually involves the modification and alteration of existing classes of antibiotics and the incorporation of synthetic products. The plant-derived chemicals display a wide variety of functional groups and chirality that can be harvested to produce novel and applicable courses of antimicrobial treatments. The results of the study present demonstrate that *Plectranthus amboinicus* is a potential source of such antimicrobial agents, namely, cell permeability enhancers, efflux pump inhibitors, and target bacterial antioxidant systems. With such a multimodal mechanism of antibacterial activity, leaf extracts of this species are also less likely to develop resistance in comparison with conventional antibiotics.

## Figures and Tables

**Table 1:** Effect of Indian borage extract on the catalase activity in *P. aeruginosa*.

Samples	Height of Foam (cm)	Percentage Change
Untreated culture control	2.5	-
Ciprofloxacin	0.3	88%
Indian borage	1	60%
1% DMSO	2	20%

**Table 2:** Effect of Indian borage extracts on the catalase activity in *S. aureus*.

Samples	Height of Foam (cm)	Percentage Change
Untreated culture control	1	-
Ciprofloxacin	No foam	-
Indian borage	0.5	50%
1% DMSO	1	-

**Table 3:** Effect of Indian borage extract on lipid peroxidation in *P. aeruginosa* and *S. aureus*.

Samples	Mean Concentration of Malondialdehyde [nm] (% Change in Lipid Peroxidation)	Std.dev	*p* Value	Mean Concentration of Malondialdehyde [nm] (% Change in Lipid Peroxidation)	Std.dev	*p* Value
	*P. aeruginosa*			*S. aureus*		
Untreated	571	0.00057	-	256.2	0	-
1% DMSO	560 (↓ 2%)	0.0006	*** *p* < 0.001	230.6 (↓ 10%)	0.0065	*** *p* < 0.001
Ciprofloxacin	864 (↑ 51.31%)	0.00057	*** *p* < 0.001	403.5 (↑ 57%)	0.0015	*** *p* < 0.001
IB	813 (↑ 42.38%)	0.00023	*** *p* < 0.001	365.1 (↑ 42.5%)	0.0005	*** *p* < 0.001

The *p* value designated as *** represents high significance. The upward and downward arrows represent increase and decrease in Malondialdehyde concentrations.

## Data Availability

All the data has already been provided in the paper and the supplementary section.

## Supplementary Materials

The following supporting information can be downloaded at: https://www.mdpi.com/article/10.3390/pathogens12060853/s1. Table S1.1: Effect of Indian borage extracts on lipid peroxidation in *P. aeruginosa*; Table S1.2: Anova table for the effect of Indian borage extracts on lipid peroxidation in *P. aeruginosa*; Table S1.3: The effect of Indian borage extracts on lipid peroxidation in *S. aureus*; Table S1.4: Anova table for the effect of Indian borage extracts on lipid peroxidation in *S. aureus*; Table S2.1: Effect of IB extracts on reactive oxygen species in *P. aeruginosa*; Table S2.2: Anova table of the effect of IB extracts on ROS in *P. aeruginosa*; Table S2.3: Effect of IB extracts on reactive oxygen species in *S. aureus*; Table S2.4: Anova table of the effect of IB extracts on ROS in *S. aureus*; Table S3.1: Effect of IB extracts on cytoplasmic membrane permeability in *P. aeruginosa*; Table S3.2: Effect of IB extracts on cytoplasmic membrane permeability in *S. aureus*; Effect of IB extracts on efflux pumps in *P. aeruginosa* and *S. aureus* using R-6-G uptake assay; Table S4.2: Anova table of the effect of IB extracts on efflux pumps in *P. aeruginosa* using R-6-G uptake assay.
