# Supplementary material for: Evaluation of the Effect of Plectranthus amboinicus L. Leaf Extracts on the Bacterial Antioxidant System and Cell Membrane Integrity of Pseudomonas aeruginosa PA01 and Staphylococcus aureus NCTC8325
Source: Pathogens. 2023 Jun 20;12(6):853. doi: 10.3390/pathogens12060853 (PMC10302856; doi:10.3390/pathogens12060853)
Supplement: Supplementary file 1 [file pathogens-12-00853-s001.zip › pathogens-2432086-supplementary.pdf]

1. Lipid peroxidation

Table S1.1: Effect of Indian borage extracts on lipid peroxidation in *P. aeruginosa*

Samples	Absorbance at 535nm	Abs-blank	Concentration of malondialdehyde [mM]	Concentration of malondialdehyde [µM]	Concentration of malondialdehyde [nM]	% Change in lipid peroxidation	S. D
Untreated	0.125	0.089	0.000571	0.571	571	-	0.00057
1% DMSO	0.122	0.086	0.0005606	0.56	560	Decrease by 2%	0.0006
Cipro	0.135	0.135	0.000864	0.864	864	Increase by 51.31%	0.00057
IB	0.127	0.127	0.000813	0.813	813	Increase by 42.38%	0.00023
Blank	0.036	-					

Table S1.2 Anova table for the effect of Indian borage extracts on lipid peroxidation in *P. aeruginosa*

Comparison	Mean Difference	q	P value
Untreated vs 1%DMSO	11.00	36666	*** P<0.001
Untreated vs Ciprofloxacin	-293.00	976641	*** P<0.001
Untreated vs IB	-242.00	806645	*** P<0.001
1%DMSO vs Cipro	-304.00	1013307	*** P<0.001
1%DMSO vs IB	-253.00	843311	*** P<0.001
Cipro vs IB	51.00	169996	*** P<0.001

Table S1.3 The effect of Indian borage extracts on lipid peroxidation in *S. aureus*

Samples	Absorbance at 535nm	Abs-blank	Concentration of malondialdehyde [mM]	Concentration of malondialdehyde [µM]	Concentration of malondialdehyde [nM]	% Change in lipid peroxidation	S. D
Untreated	0.076	0.04	0.0002562	0.2562	256.2	-	0
1% DMSO	0.072	0.036	0.0002306	0.2306	230.6	Decrease by 10%	0.0065
Cipro	0.099	0.063	0.0004035	0.4035	403.5	Increase by 57%	0.0015
IB	0.093	0.057	0.0003651	0.3651	365.1	Increase by 42.5%	0.0005
Blank	0.036	-					

Table S1.4 Anova table for the effect of Indian borage extracts on lipid peroxidation in *S. aureus*

Comparison	Mean Difference	q	P value
Untreated vs 1%DMSO	25.600	13257	*** P<0.001
Untreated vs Ciprofloxacin	-147.30	76278	*** P<0.001
Untreated vs IB	-108.90	56393	*** P<0.001
1%DMSO vs Cipro	-172.90	89534	*** P<0.001
1%DMSO vs IB	-134.50	69649	*** P<0.001
Cipro vs IB	38.400	19885	*** P<0.001

2. Reactive oxygen species

Table S2.1: Effect of IB extracts on reactive oxygen species in *P. aeruginosa*.

Samples (AU·CFU mL−1)	Untreated	1%DMSO	Cipro	IB
Average	28975	32225	204943.6	45060.6
S. D	414.07	1035.1	14298	4222.4

Table S2.2 Anova table of the effect of IB extracts on ROS in *P. aeruginosa*.

Comparison	Mean Difference	q	P value
Untreated vs 1%DMSO	-3250	0.7531	ns P>0.05
Untreated vs Ciprofloxacin	-175969	40.774	*** P<0.001
Untreated vs IB	-160869	44.727	*** P<0.001
1%DMSO vs Cipro	-172719	40.021	*** P<0.001
1%DMSO vs IB	-12836	2.974	ns P>0.05
Cipro vs IB	1598883	37.047	*** P<0.001

Table S2.3: Effect of IB extracts on reactive oxygen species in *S. aureus*.

**Samples (AU ·CFU mL−1)**	**Untreated**	**1%DMSO**	**Cipro**	**IB**
**Average**	27375.33	34006.3	186044.3	151678.6
**S.D.**	4830.1	86.8	5848.4	1155.5

Table S2.4 Anova table of the effect of IB extracts on ROS in *S. aureus.*

**Comparison**	**Mean Difference**	**q**	**P value**
Untreated vs 1%DMSO	-6631	2.994	ns P>0.05
Untreated vs Ciprofloxacin	-158669	71.633	*** P<0.001
Untreated vs IB	-124303	56.118	*** P<0.001
1%DMSO vs Cipro	-152038	68.639	*** P<0.001
1%DMSO vs IB	-117672	533.124	ns P>0.05
Cipro vs IB	34366	15.515	*** P<0.001

3. Evaluation of cell membrane integrity as a potential antimicrobial target

a. Cytoplasmic membrane permeability assay

Table S3.1: Effect of IB extracts on cytoplasmic membrane permeability in *P. aeruginosa.*

	**Absorbance (nm)**							**% dye released**
**SAMPLES**	**0**	**10**	**20**	**30**	**40**	**50**	**60**	
**Untreated**	0.035	0.035	0.045	0.055	0.068	0.095	0.095	-
**1% DMSO**	0.043	0.044	0.115	0.115	0.118	0.119	0.138	45%
**Cipro**	0.044	0.044	0.109	0.121	0.134	0.141	0.162	71%
**20% acetone**	0.038	0.039	0.044	0.05	0.067	0.078	0.099	4.2%
**IB**	0.043	0.044	0.115	0.115	0.132	0.149	0.15	58%

Table S3.2: Effect of IB extracts on cytoplasmic membrane permeability in *S. aureus.*

	**Absorbance (nm)**							**% dye released**
**SAMPLES**	**0**	**10**	**20**	**30**	**40**	**50**	**60**	
**Untreated**	0.032	0.039	0.039	0.045	0.070	0.089	0.099	-
**1% DMSO**	0.043	0.044	0.046	0.055	0.103	0.114	0.158	59%
**Cipro**	0.031	0.036	0.089	0.125	0.138	0.147	0.178	78%
**20% Acetone**	0.036	0.037	0.040	0.042	0.055	0.068	0.086	-
**IB**	0.040	0.040	0.094	0.114	0.179	0.179	0.181	83%

b. Rhodamine-6-G uptake assay

Table S4.1: Effect of IB extracts on efflux pumps in *P. aeruginosa* and *S. aureus* using R-6-G uptake assay.

	Absorbance at 527nm (% inhibition)	
**Samples**	*P. aeruginosa*	*S. aureus*
Untreated	0.2	0.63
Reserpine	0.095 (52.5%)	0.141 (77%)
1%DMSO	0.271	0.65
Cipro	0.129 (35.5%)	0.328 (48%)
IB	0.149 (25.5%)	0.477 (24.2%)

Table S4.2: Anova table of the effect of IB extracts on efflux pumps in *P. aeruginosa* using R-6-G uptake assay.

Comparison	Mean Difference	q	P value
Untreated vs reserpine	0.1045	16.154	*** P<0.001
Untreated vs 1% DMSO	-0.7125	11.014	ns P>0.05
Untreated vs Cipro	0.07050	10.898	*** P<0.001
Untreated vs IB	0.05800	8.966	*** P<0.001
Reserpine vs 1%DMSO	-0.1758	27.169	*** P<0.001
Reserpine vs Cipro	-0.03400	5.256	* P<0.05
Reserpine vs IB	-0.04650	7.188	**P<0.001
1%DMSO vs Cipro	0.1418	21.913	*** P<0.001
1%DMSO vs IB	0.1293	19.980	*** P<0.001
Cipro vs IB	-0.01250	1.932	ns P>0.05

Table S4.3: Anova table of the effect of IB extracts on efflux pumps in *S. aureus* using R-6-G uptake assay.

Comparison	Mean Difference	q	P value
Untreated vs reserpine	0.4890	16.033	*** P<0.001
Untreated vs 1% DMSO	-0.01950	0.6384	ns P>0.05
Untreated vs Cipro	0.3025	9.918	*** P<0.001
Untreated vs IB	0.1533	5.025	* P<0.05
Reserpine vs 1%DMSO	-0.5085	16.673	*** P<0.001
Reserpine vs Cipro	-0.1865	6.115	** P<0.01
Reserpine vs IB	-0.3358	11.009	***P<0.001
1%DMSO vs Cipro	0.3220	10.558	*** P<0.001
1%DMSO vs IB	0.1728	5.664	** P<0.01
Cipro vs IB	-0.1493	4.894	* P<0.05
